# Salt Stress Induces Changes in Physiological Characteristics, Bioactive Constituents, and Antioxidants in Kenaf (*Hibiscus cannabinus* L.)

**DOI:** 10.3390/antiox11102005

**Published:** 2022-10-10

**Authors:** Ziggiju Mesenbet Birhanie, Dawei Yang, Mingbao Luan, Aiping Xiao, Liangliang Liu, Chao Zhang, Ashok Biswas, Susmita Dey, Yong Deng, Defang Li

**Affiliations:** 1 Institute of Bast Fiber Crops, Chinese Academy of Agricultural Sciences, Changsha 410205, China; 2 Department of Plant Science, College of Agriculture and Natural Resources, Debre Markos University, Debre Markos 251269, Ethiopia

**Keywords:** *Hibiscus cannabinus*, salt stress, physiological changes, bioactive constituents

## Abstract

Salinity stress is a major environmental threat in agricultural systems. Kenaf is a promising crop for the future for cultivation in salinity-affected soils because of its high phytoremediation potential. The current study aimed to investigate the effects of salt stress using six different sodium chloride (NaCl) concentrations (0, 50, 100, 150, 200, and 250 mM) on the plant growth, physiological characteristics, bioactive constituents, and antioxidant capacity of *H. cannabinus*. The results indicated that the NaCl stress induced significant reductions in plant height and in the dry and fresh weights of the leaf tissue. In addition, the K, Ca, Mg, and P concentrations in this tissue also decreased under NaCl stress treatment conditions. In contrast, the NaCl stress led to the accumulation of hydrogen peroxide (H_2_O_2_), superoxide anion (O_2_^•−^), malondialdehyde (MDA), proline, total soluble sugar, and total soluble protein. Under NaCl stress, the levels of antioxidants, including phenolics and flavonoids, also increased. The gas chromatography–mass spectrometry (GC-MS) results showed that the volatile compounds, including heptacosane, 1-octadecanesulphonyl chloride, and tetratetracontane, were induced under the NaCl stress treatment. Furthermore, the salt stress significantly improved the antioxidant capacity of the leaf extracts. These findings may provide insight into how *H. cannabinus* plants respond to salt stress and may help improve its medicinal value under salt stress.

## 1. Introduction

Salinity is among the major abiotic stresses impacting plant growth and productivity [1,2]. It affects approximately 1125 million hectares of agricultural land globally [3]. In China, salinity affects about 36.7 million hectares of land. By 2050, it could damage more than 50% of the agricultural land [4]. The intensity of the salt stress affects the plants’ morphological, physiological, and metabolic changes. Soil salinity can inhibit plant growth by causing ion toxicity, osmotic and oxidative stresses, pigment degradation, and photosynthesis inhibition [5,6,7]. Ion toxicity and osmotic stress cause nutritional imbalances and oxidative stress by restricting plants from extracting water from the soil and from inside the plants themselves [8,9]. Additionally, salt stress causes oxidative stress by increasing reactive oxygen species (ROS), such as hydrogen peroxide (H_2_O_2_), superoxide (O_2_**^•^**^−^), and hydroxyl radicals (OH**^·^**) [10,11]. Salt stress can severely disrupt the equilibrium between producing and scavenging reactive oxygen species (ROS) [12]. Plants require a certain threshold level of reactive oxygen species (ROS) to function normally; any variation in the ROS concentration can have detrimental effects on a plant’s physiology [13]. Specifically, excessive concentrations of radical species cause damage to plant cell components, resulting in cell death [14].

Plants have varied defense strategies against salt stresses, involving morphological, physiological, and molecular responses [15]. Plants can produce osmolytes, including soluble sugars, proteins, and proline, which protect plant cells from the adverse effects of salt stress [16,17]. Protecting cellular membranes via enzymatic antioxidants against salt-induced ROS over-production and membrane lipid peroxidation leads to salt tolerance [18,19,20]. Under salt stress, antioxidants such as phenolic and flavonoid compounds might also act as ROS scavengers [21]. Plants also defend themselves against biotic and abiotic stressors by emitting volatile organic molecules [22]. Multiple classes of terpenes, phenylpropanoids, and benzenoids, as well as volatile fatty acid and amino acid derivatives, are among the volatile chemicals produced in response to stress [23]. Moreover, salt-stressed plants regulate salt-stress-related genes and have signal transduction factors [24].

Kenaf (*Hibiscus cannabinus* L.) is a major annual fiber crop native to east-central Africa and widely grown in the Asia–Pacific region. *Hibiscus cannabinus* cultivation has increasingly shifted to saline land due to an increased demand for food crops and reduced available arable land [25]. Although kenaf is mostly used for fiber, the seeds, leaves, and flowers may be useful in the food industry [26]. It is also used as a cosmetic ingredient and in folk medicine. *Hibiscus cannabinus* contains bioactive components such as phenolics, flavonoids, terpenes, citric acid, and fatty acid derivatives, which have a variety of pharmacological activities. For example, phenolic compounds have antiaging [27], antiproliferative [28], antityrosinase [29], and antioxidant properties [30]. Flavonoid-rich products also have a variety of biological actions, such as antibacterial [30], anti-inflammatory [31], antioxidant [32], and antidiabetic activities [33]. Moreover, phytol, an acyclic diterpene alcohol, can be used as a precursor in producing synthetic vitamins E and K1 [34]. Hydroxycitric acid has been demonstrated to lower blood insulin levels [35]. Omega-3 polyunsaturated fatty acids are also responsible for lowering the risk of cardiovascular disease and the fracture risk [36]. The essential oil composition and phytotoxic and fungitoxic activity levels of kenaf leaves were investigated by Kobaisy et al. [22]. The oil was effective against *Colletotrichum gloeosporioides*, *Colletotrichum fragariae*, and *Colletotrichum accutatum*, while also being phytotoxic to bentgrass and lettuce. In addition, aqueous extracts of kenaf leaves have been shown to protect rats’ livers from carbon tetrachloride and paracetamol-induced damage [37]. Diet-induced hyperlipidemia was mitigated by a hydroalcohol extract of *H. cannabinus* leaves [38]. Kenaf extract induces a cytoprotective molecule in activated macrophages, resulting in a significant immunomodulatory effect [39]. Secondary metabolites of *H. cannabinus*, namely phenolics, flavonoids, and phenolic acids, correlate strongly with the antioxidant capacity, and these compounds prevent oxidative damage to cells by lowering ROS levels under salt stress [40].

Most previous studies have concentrated on the phytochemical properties of *H. cannabinus* under normal conditions [32,41]. However, the acumination and synthesis of bioactive and nutritional compositions depend on abiotic stresses [16,42]. Many plants subjected to salinity stress exhibit changes in the composition of the phenolics [43], flavonoids [44], and saponins [45]. These alterations are dependent on the degree and duration of the stress. Thus, plants stressed by salinity may have the potential to be polyphenol sources. Furthermore, *Hibiscus cannabinus* can potentially be used in phytoremediation to remediate salt-affected soils due to its suitability for cultivation in salinity-affected soils [46,47]. Despite these promising features, the physiological and biochemical responses of *H. cannabinus* to salinity conditions have been scarcely studied. The current study was, therefore, carried out to investigate the effects of six different salt concentrations (0, 50, 100, 150, 200, and 250 mM of NaCl) on the plant growth, physiological traits, bioactive components, and antioxidant capacity of *H. cannabinus*.

## 2. Materials and Methods

### 2.1. Plant Material, Growth Conditions, and Salt Treatments

China kenaf 21, a typical kenaf variety from the Institute of Bast Fiber Crops, Chinese Academy of Agricultural Sciences, was chosen for this study. The kenaf seeds were placed on moist filter paper and were enabled to germinate for three days at a temperature of 25 °C in the dark after being soaked in sterile water for five hours. The germinated seeds were transferred to a ¼-strength Hoagland nutrient solution (pH 6.0) comprising 5.79 mmol L^−1^ Ca (NO_3_), 2, 4.17 mmol L^−1^ MgSO4, 8.90 mol L^−1^ MnSO_4_, 8.02 mmol L^−1^ KNO_3_, 0.94 mol L^−1^ ZnSO_4_, 1.35 mmol L^−1^ NH_4_H_2_PO_4_, 0.20 mol CuSO_4_, 0.015 µmol L^−1^ (NH_4_)_2_MoO_4_, 48.3 µmol L^−1^ H_3_BO_3_, and 72.6 µmol L^−1^ Fe-EDTA for continued growth [48]. After 5 days, the seedlings were transferred to a ¼-strength Hoagland nutrient solution supplemented with 0 (control), 50, 100, 150, 200, and 250 mm NaCl solutions, with three replicates at each concentration level, replenished every two days. The seedlings were grown in a culture chamber with a 28/25 °C temperature regime, a photoperiod of 16 h/8 h (light/dark), relative humidity of around 60%, and a light intensity of 700 µmol m^−2^s^−1^. The plants were harvested 14 days after being subjected to salt stress because the plants treated with 200 and 250 mM of NaCl showed salt stress symptoms (leaf chlorosis and necrosis).

### 2.2. Chemicals and Reagents

Solar Bio-Science and Technology Co. (Beijing, China) supplied the 2,2-diphenyl-1-picrylhydrazyl (DPPH), 2,2′-azinobis (3-ethylbenzothiazoline-6-sulfonic acid (ABTS), rutin, and gallic acid. Coolaber Technology Co. (Beijing, China) provided the vanillin, 2,4,6-tripyridyl-s-triazine (TPTZ), and Folin–Ciocalteau reagent. The ferrozine, iron sulfate heptahydrate, and other chemicals were purchased from Shanghai Macklin Biochemical Technology Co. (Shanghai, China). All reagents used in the assay were of the highest analytical grade.

### 2.3. Parameters for Plant Growth

The plant height and fresh weight (FW) values were measured fourteen days after saline treatment. The plant material was oven-dried to a constant weight at 50 °C and the dry weight (DW) was recorded.

### 2.4. Determination of Mineral Contents

The plant leaves were powdered to a fine powder using a small crusher after drying for five days at 50 °C. In a 50 mL crucible, 0.1 g of each sample powder was digested with 5 mL of concentrated HNO_3_ and 1 mL of HClO_4_ (70%). The mixture was then heated to 150–200 °C on a hot plate until the digest became semi-dried. The cooled sample was dissolved in deionized water to a total volume of 20 mL before the analysis [49,50].

The content of K was measured using a flame photometer (Model: FP6431, Shanghai Yidian Analysis Instrument Co., Ltd., Shanghai, China). The Ca and Mg contents were determined using an atomic absorption spectrophotometer (Model: 3110, Thermo Scientific, Oxford, UK). The concentrations of P^3+^ were determined using UV–Vis spectrophotometry (UV-2007, Shimadzu Global Laboratory Consumables Co., Ltd., Shanghai, China). Inductively coupled plasma mass spectrometry (iCAP Q ICP-MS, Thermo Fisher Scientific, Germany) was also used to determine the content of Fe^2+^. The ICP-MS operating conditions were as follows: the radio frequency (R.F.) power was 1550 W, the nebulizer gas flow was 1.01 L/min, the auxiliary gas flow was 0.8 L/min, and the cool gas drift was 14 L/min.

### 2.5. Quantification of Proline, Total Soluble Sugar, and Protein Contents

The Bates method, with minor modifications, was used for proline determinations [51]. The fresh leaves (0.5 g) were homogenized in 5 mL of sulfosalicylic acid (3%) and incubated at 100 °C for 10 min. The supernatant (2 mL) was mixed with 2 mL of ninhydrin reagent and 2 mL of glacial acetic acid. The mixture was then allowed to cool to room temperature and centrifuged for 10 min at 3000 rpm. The mixture was incubated at 100 °C for 1 h before being cooled in an ice bath for 15 min. As a reaction reagent, 4 mL of toluene was added to the previous mixture, and the absorbance at 520 nm was measured. The proline content was determined using the standard curve and expressed as g g^−1^ FW.

The total soluble sugars were determined using the phenol–sulfuric acid method [52]. The fresh samples (0.5 g) were homogenized in 10.0 mL of 80% ethanol and centrifuged for 20 min at 2000 rpm, then the supernatant was collected. The supernatant (0.1 mL) was mixed with 1.0 mL phenol (5%) and 5.0 mL of sulfuric acid (98%). The mixture was then allowed to stand in a 30 °C water bath for 20 min. Finally, the absorbance was measured at 490 nm. The amount of available soluble sugar was calculated using a glucose calibration curve (10–100 mg/mL) and expressed as mg/g FW.

The soluble protein was measured using the method used by Guy [53]. The fresh samples (0.5 g) were homogenized in a solution of 50 mM Tris-HCl (pH 7.5), 2 mM EDTA, and 0.04% (*v/v*) β-mercaptoethanol and centrifuged at 10,000× *g* for 15 min. After mixing 1 mL of supernatant with 1 mL of Coomassie Brilliant Blue, the absorbance was read at 595 nm.

### 2.6. ROS Determination—Hydrogen Peroxide (H_2_O_2_) and Superoxide Anion (O_2_^•−^)

The H_2_O_2_ content was determined using the method developed by Okuda et al. [54]. The fresh leaf samples (200 mg) were milled in 2 mL of 200 mM perchloric acid in an ice bath and then centrifuged for 10 min at 12,000× *g*. After centrifugation, 4 M KOH was used to neutralize the perchloric acid in the supernatant. The insoluble potassium perchlorate was then removed by centrifugation at 500× *g* for 3 min. The supernatants (1 mL) were combined with 400 µL of 3-(dimethylamino) benzoic acid (12.5 mM) in phosphate buffer (0.375 M, pH 6.5), 80 µL of 3-methyl-2-benzothiazoline hydrazone, and 20 µL of peroxidase (0.25 unit). The reaction was started by adding peroxidase at 25 °C, then the absorbance was measured at 590 nm.

The superoxide radical (O_2_^•−^) content was determined using the techniques used by Bu et al. [55] and Lang et al. [56] with minor modifications. The fresh leaf samples (0.2 g) were treated for 1 h with 1 mL of hydroxylamine hydrochloride. The mixture was then incubated at 25 °C for 20 min with 1 mL each of α-naphthylamine and 2-aminobenzenesulfonic acid. The absorbance of the solution at 530 nm was measured. The O_2_^•−^ content was calculated using a NaNO2 calibration curve (10–100 mg/mL).

### 2.7. Measurement of the Lipid Peroxidation–MDA Content

The MDA content was determined using Heath and Packer’s technique [57]. Roughly 0.5 g fresh leaf samples were pulverized in 5 mL of trichloroacetic acid (10%) and 2-thiobarbituric acid (0.65%). The mixture was then heated for 1 h at 100 °C, cooled to ambient temperature, and centrifuged at 10,000 rpm for 10 min, then the absorbances of the samples were measured in triplicate at 532, 600, and 450 nm. The MDA content of the reaction solution was determined using the following equation:

MDA (µmol g − 1 FW) = 6.45 (A532 − A600) − (0.56 A450)(1)

The MDA concentration was calculated using an extinction coefficient of 155 mmol L^−1^ cm^−1^ and expressed as nmol of MDA g^−1^ FW.

### 2.8. Preparation of Ethanol Extracts

The collected leaves were vacuum-dried at 40 °C. The dried samples were ground into a powder using a small crusher (HX-200 A, Xian Hardware and Pharmacy Co., Ltd., Xian, China). For each treatment, 1 g of powdered leaves was extracted ultrasonically for 30 min with a 10 mL ethanol solution (80%). The mixtures were centrifuged for 3 min at 4 °C at 4000× *g* [58]. The supernatant was collected and stored at 4 °C for 48 h.

### 2.9. Determination of Total Phenolic and Flavonoid Contents

The phenolic content was determined using a Folin–Ciocalteau colorimetric assay [41]. Briefly, 1.5 mL of 20% (*v*/*v*) Folin–Ciocalteau reagent was mixed thoroughly into 0.2 mL of sample extract and allowed to stand for 5 min. The total volume was then built up to 10 mL with distilled water before being incubated in the dark for 90 min at ambient temperature. The absorbance was measured at 760 nm against a prepared blank. The calibration curve of gallic acid, which ranged from 20 to 800 mg/L, was used to determine the phenolic content. The results are presented in milligrams of equivalent gallic acid per gram of sample dry weight (mg GAE/g DW).

The flavonoid content of each extract was determined using the aluminum chloride technique [32]. First, 0.2 mL of sample extract was mixed with 4 mL of distilled water, followed by 0.3 mL of NaNO_2_ (5%), and reacted for 5 min. After this, 0.3 mL of AlCl_3_ (10%) was added and left to react for 6 min. Then, after adding 2 mL of NaOH (4%) and filling it with up to 10 mL of distilled water, the absorbance of the solution was measured at 510 nm. The flavonoid content was determined using a rutin standard curve ranging from 50 to 400 mg/L. The results are given as milligrams of rutin equivalents (mg RE/g DW) for each gram of dry weight of the samples.

### 2.10. Determination of Total Saponin Content

The saponin content of the plant extract was determined using the vanillin–sulfuric acid colorimetric method [59]. In brief, 0.1 mL of sample extract was mixed with 0.5 mL of ethanol (50%), 0.5 mL of vanillin solution (8%), and 4.0 mL of sulfuric acid (77.5%). The solution was then cooled to room temperature after 15 min incubation in a water bath at 60 °C. Then, the absorbance was measured using a spectrophotometer at 545 nm. The results were expressed as milligrams of tea saponin equivalents per gram of dry weight of the sample (TSE/g DW) using a calibration curve ranging from 50 to 400 mg/L.

### 2.11. Gas Chromatography–Mass Spectrometry (GC–MS) Identification of Volatile Compounds

The plant extraction for the GC-MS analysis was performed as previously described [32]. Briefly, 2.5 g of the powdered leaf was extracted in 50 mL of hexane using ultrasonic-assisted extraction for 30 min for each treatment. The extracts were purified (Whatman no. 4), evaporated at reduced pressure and temperature using a rotary evaporator, weighed, and dissolved in hexane at 10 mg/mL. The samples were analyzed via GC-MS equipped with an HP-5ms capillary column (30 m × 0.25 mm × 0.25 μm). The carrier gas was pure helium with a purity greater than 99.99%, flowing at a rate of 1.2 mL/min. The sample was diluted with n-hexane at a 10% (*v*/*v*) concentration. The injection volume was 1 µL and the diversion ratio was 1:5. The temperatures for injection and detection were set to 250 and 280 °C, respectively. The chromatographic heating procedure was as follows: the temperature was initially set to 60 °C for 2 min, then raised to 280 °C at a rate of 5 °C/min for 9 min. The electron ionization mode of the mass spectrometry involved an electron energy of 70 eV, a scanning range of 40–400 (*m/z*), a scanning rate of 3.99 scans/s, and a solvent delay of 3 min. The retention time (RT) values and NIST05 mass spectral library were used to identify compounds. The relative peak area of each compound in the chromatogram was used to calculate the percentage of each compound.

### 2.12. Detection of Antioxidant Activity

The DPPH• scavenging assay was performed with minor modifications to the Brand–Williams method [60]. A DPPH solution in methanol (6 × 10^−5^ M) was prepared and mixed with 100 µL of each sample (3 mL). The sample absorbance (A1) was measured at 515 nm after the mixtures were incubated in the dark for 15 min at room temperature. The absorbance of a blank sample (A0) containing 100 µL of methanol was also measured. The scavenging ability of the triplicate experiments was estimated using the following equation:

Inhibition (%) = [(A0 − A1)/A0] × 100(2)

where A0 is the absorbance of the blank and A1 is the absorbance of the sample extract.

The ABTS assay was measured following the procedure described by Nisca et al. [61]. Furthermore, 100 µL of sample extract was mixed with 100 µL of ABTS reagent and left to react in the dark for 6 min. The absorbance of the sample was measured at 734 nm. The inhibition percentage was calculated using the above formula described in the DPPH method.

The FRAP was determined using a slightly modified Benzie and Strain [62] method. The fresh FRAP reagent working solution was prepared by mixing 20 mL of acetate buffer (300 mM, pH 3.6), 2 mL of TPTZ (10 mM) in 40 mM HCl, and 2 mL of FeCl_3_, 6H_2_O (20 mM). The mixture was then incubated in a water bath at 37 °C for 30 min. The samples (75 µL) were then vigorously mixed with 75 mL of FRAP reagent. The sample absorption was measured at 593 nm after 4 min. A ferrous sulfate solution (0.5–10 mg/mL) was used to create the standard curve. The results were expressed in millimoles of ferrous ion equivalent per gram dry weight of the sample (mmol Fe^2+^/g DW).

The chelation ability of the ethanol extract was determined using a previously described ferrozine-based colorimetric assay [63]. The ethanol extract (50 µL) was mixed with 200 µL FeSO_4_ (0.2 mM) and 200 µL ferrozine (0.5 mM). The mixture was shaken and left at room temperature for 10 min. Finally, the absorbance was measured at 562 nm. The inhibition percentage of the ferrozine–Fe^2+^ complex was calculated using the following equation:

Inhibition (%) = [(Ac − As)/Ac] × 100(3)

where Ac is the absorbance of the control and As is the absorbance of the sample.

### 2.13. Statistical Analysis

The data for all parameters were subjected to a one-way ANOVA followed by Duncan’s multiple comparisons (*p* < 0.05) test using SAS version 9.4 (SAS Inc., Cary, NC, USA). The results are presented as mean values ± standard deviations (SD). For graphical representations, OriginPro^®^ version 9.8.0.200 software (Northampton, MA, USA) was used.

## 3. Results

### 3.1. Effect of Salt Stress on Growth Parameters

**Figure 1:**
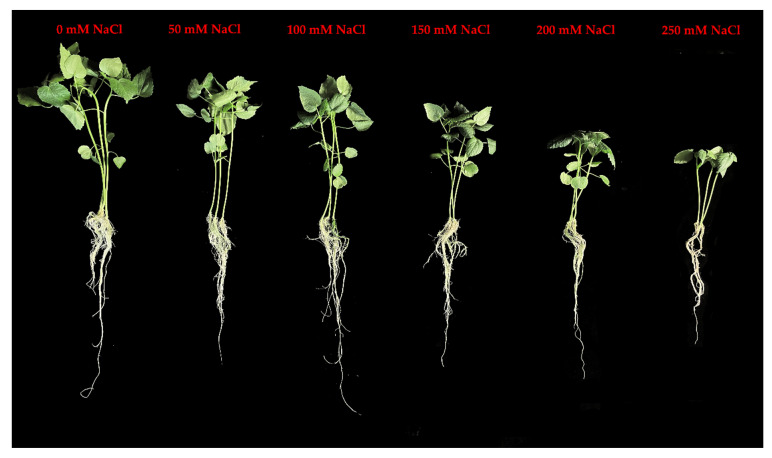
Changes in morphology of *H. cannabinus* seedlings grown under different salt stress conditions (0 mM of NaCl, 50 mM of NaCl, 100 mM of NaCl, 150 mM of NaCl, 200 mM of NaCl, and 250 mM of NaCl).

**Figure 2:**
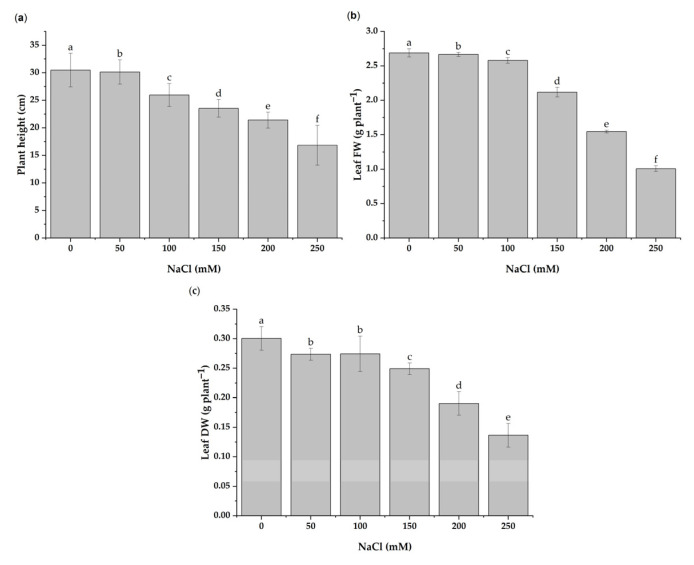
Effects of different levels of salinity stress (0, 50, 100, 150, 200, and 250 mM of NaCl) on the plant height (**a**) and fresh weight (FW) (**b**) and dry weight (DW) (**c**) of *H. cannabinus* leaves. The results are expressed in cm or g plant-1, as the means ± SD of different measurements (*n* = 15). Different letters (a–f) above the bars indicate a significant difference between treatments according to the Duncan test (*p* < 0.05).

The effect of NaCl on the *H. cannabinus* plant growth was assessed by measuring the plant height as well as the fresh and dry weights of the leaf. The results revealed that the salinity significantly decreased the plant growth, as depicted in Figure 1. In detail, the treatments with 100, 150, 200, and 200 mM of NaCl significantly reduced the plant height by 14.80%, 22.81%, 29.78%, and 44.81%, respectively, compared to the control (Figure 2a). At 200 and 250 mM NaCl concentrations, the fresh leaf weight was reduced by 42.46 and 62.51%, respectively (Figure 2b). The leaf dry weight was also affected by the stressor, decreasing by 8.96 (150 mM), 30.45 (200 mM), and 50.11% (250 mM) (Figure 2c).

### 3.2. Impact of Salt Stress on Minerals in Leaves

Salinity stress significantly impacted the mineral concentration in the *H. cannabinus* leaves (Table 1). The salinity stress (100, 150, 200, and 250 mM) decreased the N (up to 8.49, 17.76, 22.63 and 24.85%, respectively) compared with the control. However, the N content reduction was not statistically significant at 50 mM of NaCl. The concentration of K significantly decreased at 50 mM, 200 mM, and 250 mM of NaCl by 6.52%, 5.74%, and 3.87%, respectively, as compared to the control, whereas the changes were slight at 100 and 150 mM of NaCl. Moreover, the concentrations of Ca and Mg decreased as the salinity intensified. However, the Mg content decreased slightly at 50 mM of NaCl. The concentration of *p* significantly decreased under salt stress. The low saline concentration (50 mM) significantly the increased Fe concentrations, whereas the medium and high saline concentrations significantly reduced the concentrations.

### 3.3. Alterations of Proline, Total Soluble Sugar, and Soluble Protein Contents under Salt Stress

**Figure 3:**
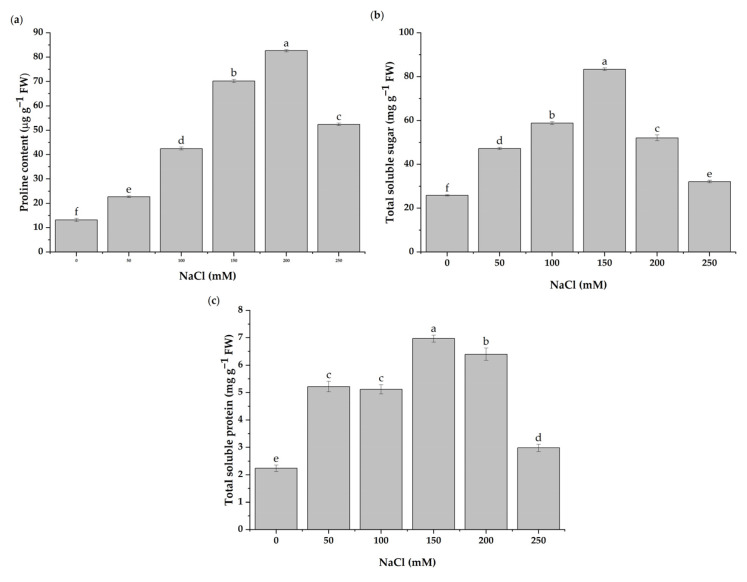
Effects of salt stress on the proline (**a**), total soluble sugar (**b**), and total soluble protein (**c**) contents in leaves of *H. cannabinus* seedlings subjected to 0, 50, 100, 150, 200, and 250 mM of NaCl. The values shown are means ± SD (*n* = 3). The different letters (a–f) above the bars indicate a significant difference according to the Duncan test (*p* < 0.05).

Proline is one of the most common osmotic adjustment substances. The results indicated that the plant proline content increased significantly with the salt concentration (Figure 3a). The proline levels increased by 432.76 and 527.38% under 150 and 200 mM NaCl concentrations, respectively, as compared with the control.

The results revealed that the NaCl concentrations increased the total soluble sugar content but to varying levels (Figure 3b). The total soluble sugar increased continuously from 50 to 150 mM of NaCl treatment. Treatments with 200 and 150 mM of NaCl increased the total soluble sugar by 101.63 and 222.59%, respectively, as compared to the control. Likewise, the application of 250 mM increased the total soluble sugar content by 24.38%, as compared to the control.

The content of soluble proteins was affected by salinity stress. The protein content increased significantly with the increasing salt concentration compared to the control (Figure 3c). The soluble protein levels were highest with 150 and 200 mM of NaCl. The soluble protein content was enhanced by 185.77 and 211.37% at 200 and 150 mM NaCl concentrations, respectively, as compared to the control treatment.

### 3.4. ROS Detection and Lipid Peroxidation

**Figure 4:**
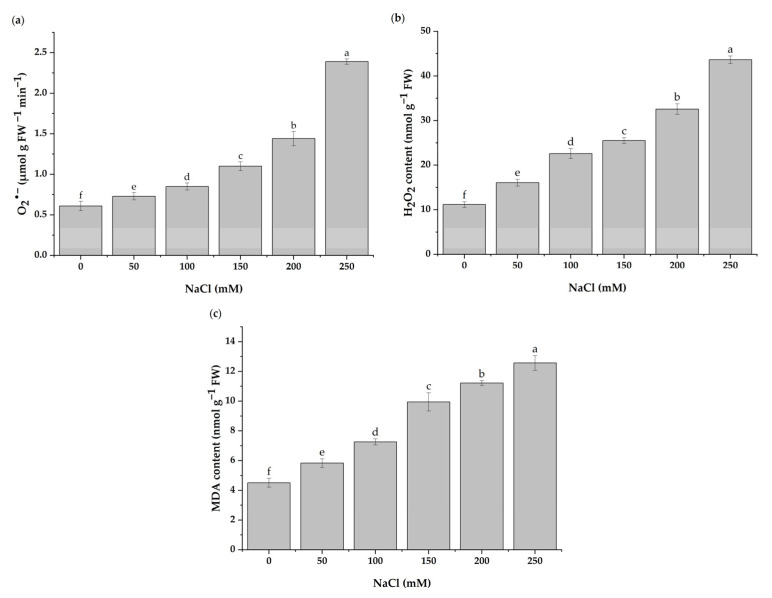
O_2_^•−^ contents (**a**), H_2_O_2_ contents (**b**), and MDA contents (**c**) in leaves of *H. cannabinus* under various salt stress levels (0, 50, 100, 150, 200, and 250 mM of NaCl). The values presented are means ± SD (*n* = 3). Different letters (a–f) above the bars indicate a significant difference between treatments according to the Duncan test (*p* < 0.05).

The concentration of O_2_^•−^ in the leaves of *H. cannabinus* was altered by the salinity. In detail, the O_2_^•−^ content significantly increased with the increasing salt concentrations, as shown in Figure 4a. The levels of O_2_^•−^ were higher in seedlings treated with 200 and 250 mM of NaCl, with the values exceeding 136.07 and 291.80% of those found in the control plants, respectively. Likewise, in seedlings exposed to 50 mM of NaCl, the O_2_^•−^ production increased by 19.67%. The H_2_O_2_ production rate increased progressively with the increase in salt concentration (Figure 4b). The H_2_O_2_ levels rose by 191.23 and 290.34% at 200 and 250 mM of NaCl, respectively, as compared to the control treatment. Moreover, 50 mM of NaCl increased the H_2_O_2_ concentration by 43.83% compared to the control.

As illustrated in Figure 4c, the MDA content is significantly influenced by the different salt concentrations. The results indicated that the MDA content increased as the salinity intensified. Compared to the control treatment, the MDA levels increased by 29.27 and 178.71% in response to 50 and 250 mM of NaCl, respectively.

### 3.5. Total Contents of Phenolics, Flavonoids, and Saponins

**Figure 5:**
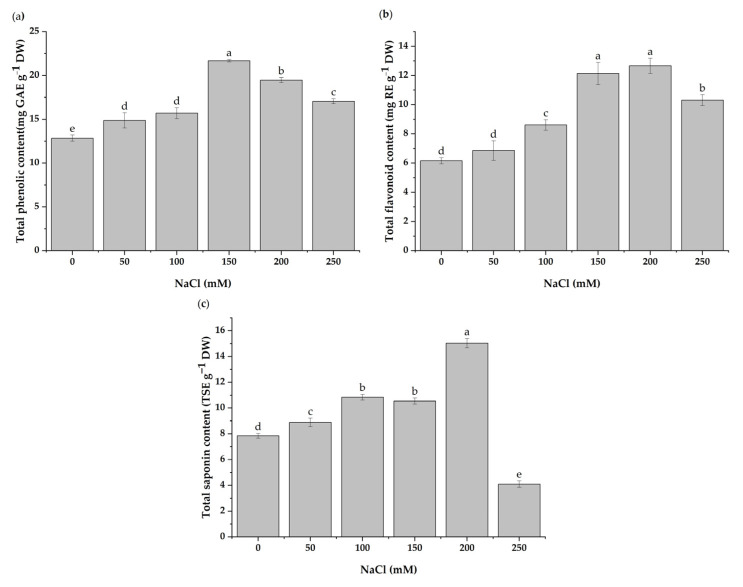
Changes in (**a**) the total phenolic contents, (**b**) total flavonoid contents, and (**c**) total saponin contents under different levels of salt stress (0, 50, 100, 150, 200, and 250 mM of NaCl). The values presented are means ± SD (*n* = 3). Different letters (a–e) above the bars indicate a significant difference between treatments according to the Duncan test (*p* < 0.05).

According to the findings, the salinity stress affected the total phenolic, flavonoid, and saponin contents. Under salt stress, the total phenolic content was found to be increased (Figure 5a). In detail, the total phenolic contents increased by 68.84 and 51.55% with 150 and 200 mM of NaCl, respectively, compared to the control. Similarly, the total flavonoid contents increased by 96.85 and 105.8% with 150 and 200 mM of NaCl, respectively, compared to the control (Figure 5b). There was no significant effect on the total flavonoid content with the 50 mM NaCl treatment. There was a considerable difference in saponin contents with increasing salt concentrations compared to the control (Figure 5c). The content of saponins increased steadily from the 50 to 200 mM NaCl treatments. Compared to the control, the saponin accumulation increased by 91.61% with 200 mM of NaCl but was significantly reduced by 47.81% with 250 mM of NaCl.

### 3.6. Alterations in the Compositions of Volatile Compounds under Salt Stress

The compositions of the leaf volatile compounds of *H. cannabinus* and their relative percentages (%) at different NaCl levels are shown in Table 2. Seventeen compounds were identified in the control. Phytol (18.64%) and 1-heptacosanol (18.23%) were the major components; other notable components were oleamide (9.36%), 12-methyl-E, E-2,13-octadecadien-1-ol (8.27%), alterungsschutzmittel BKF (7.30%), cis-vaccenic acid (6.80%), phthalic acid, hep-tyl undecyl ester (6.70%), and methyl linolelaidate (4.36%). When subjected to salt stress, the relative proportions of these components changed significantly. The application of NaCl reduced the phytol levels by 69.26%, 87.55%, and 88.79% at 150, 50, and 100 mM, respectively, compared with the control. This compound disappeared with 200 and 250 mM of NaCl. Compared to the control, the concentration of 1-heptacosanol increased significantly by 6.80% with 200 mM of NaCl; decreased substantially with 50, 100, and 250 mM of NaCl; and was not detected with 150 MM of NaCl. The biosynthesis of phthalic acid and heptyl undecyl ester was enhanced considerably with 100, 200, and 250 mM of NaCl, and it emerged as the first and most abundant compound. Also, the relative content of 3-(octadecyloxy) propyl ester increased at 50, 100, 150, and 200 mM NaCl concentrations and eventually became the second most abundant compound. The salt stress induced the biosynthesis of new compounds, including heptacosane, 1-octadecanesulphonyl chloride, and tetratetracontane. The biosynthesis of terpenes and alcohols was suppressed under salt stress. However, a high salt concentration stimulated the production of alkane. In addition, under all levels of salinity stress, the esters increased significantly and eventually became the most abundant chemical class in salt-treated plants.

### 3.7. In Vitro Antioxidant Activities under Salt Stress

**Figure 6:**
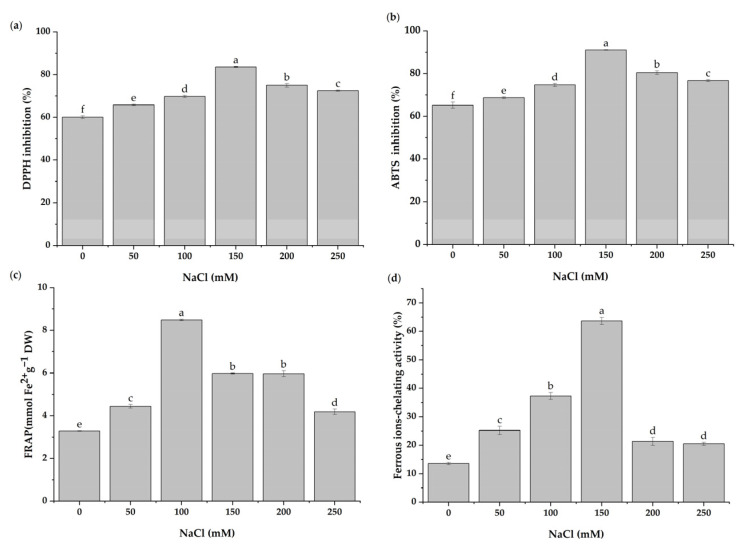
Changes in (**a**) DPPH inhibition, (**b**) ABTS inhibition, (**c**) ferrous-ion-chelating activity, and (**d**) FRAP antioxidant capacity levels in *H. cannabinus* leaves under different salt concentrations (0, 50, 100, 150, 200, and 250 mM of NaCl). The values presented are means ± SD (*n* = 3). Different letters (a–f) above the bars indicate a significant difference between treatments according to the Duncan test (*p* < 0.05).

The antioxidant activity of *H. cannabinus* extracts was determined via DPPH, ABTS, FRAP, and ferrozine assays. Under salt stress, the antioxidant activity was significantly influenced (Figure 6). The plants treated with 150 mM of NaCl showed the highest antioxidant activity levels in DPPH, ABTS, and ferrozine assays, with values of 83.60%, 91.08%, and 63.68%, respectively. The FRAP results revealed the maximum antioxidant activity in the group treated with 100 mM of NaCl, with a value of 8.49 mmol Fe^2+^/g.

**Figure 7:**
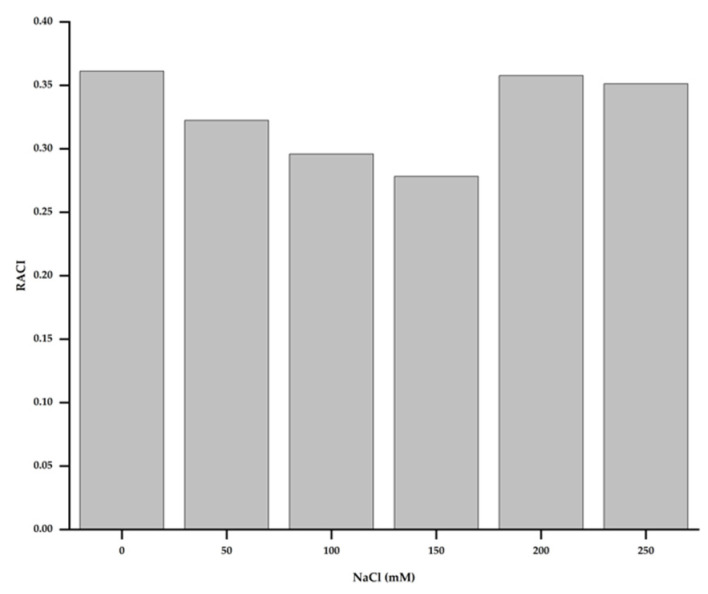
The relative antioxidant capacity index (RACI) was applied to combine the antioxidant capacity values from the various methods.

The relative antioxidant capacity index (RACI) values were calculated by the merging antioxidant capacity values from different chemical methods to rank the samples’ antioxidant capacities. The RACI values were calculated using the method described by Marić et al. [29] previously. The RACI is the mean value of transformed standard scores derived from initial data without unit or method restrictions. As shown in Figure 7, the group treated with 200 mM of NaCl exhibited the highest RACI value (0.36), followed by the group treated with 250 mM of NaCl (0.35). The lowest RACI value was observed at 150 mM of NaCl.

### 3.8. Correlation Analysis of Physiological and Biochemical Characteristics

**Figure 8:**
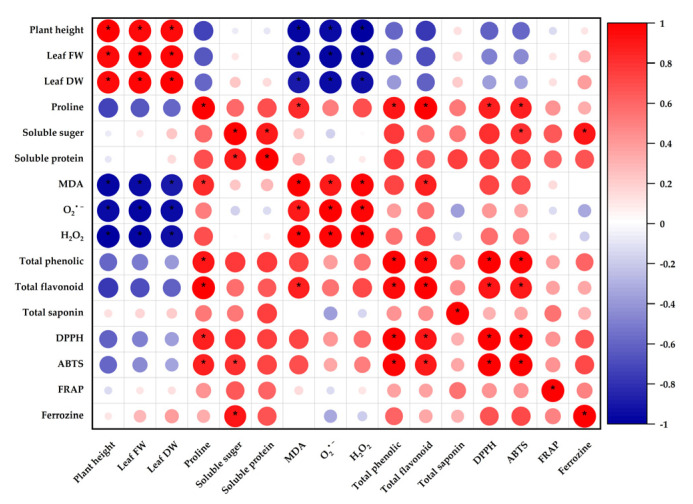
A correlation analysis of physiological, biochemical, and antioxidant activities of *H. cannabinus* seedlings under salt stress. * Indicates significance at the 5% level, while color depth denotes the correlation coefficient. The strength of the correlation is represented by the size of the circles.

The Pearson’s correlations between physiological and biochemical character traits are illustrated in Figure 8. The DPPH and ABTS scavenging capacity levels correlated positively with the proline, total phenolic, and total flavonoid contents. In addition, soluble sugar was positively correlated with the ABTS scavenging capacity. MDA showed a significant positive correlation with the reactive oxygen species (O_2_^•−^ and H_2_O_2_), proline, and total flavonoid contents. However, MDA was negatively correlated with the plant height and fresh and dry weight of the leaves.

## 4. Discussion

Soil salinization is now a major environmental threat to the long-term growth of global agriculture. It induces alterations in many physiological and metabolic processes, eventually reducing the crop yield, depending on the severity and duration of the stress [64,65]. Plants can tolerate or avoid saline conditions [65]. This study examined the growth parameters, physiology characteristics, bioactive constituents, and antioxidant capacity of *H. cannabinus* to assess its ability to deal with salinity.

The salt stress inhibited the *H. cannabinus* growth regarding the plant height and the fresh and dry weights of the leaves. Several authors have reported that various levels of salinity stress reduce plant growth in other medicinal plants [43,66,67]. In saline soils, the inhibition of plant growth is primarily caused by osmotic stress, which reduces the absorption of essential macro- and micronutrients [66]. In the current study, the salt stress decreased the concentrations of N, K, Ca, Mg, and P. However, there were no noticeable changes in the N and Mg contents with 50 mM of NaCl and only a slight change in the K content with 100 and 150 mM of NaCl. As previously demonstrated, the decreases in these minerals may be directly related to increased Na uptake by the roots [66]. Moreover, it has been found that NaCl treatment reduces Ca and Mg concentrations in plants [6,43]. However, adequate K, Ca, and Mg are needed to perform fundamental metabolic functions such as cellular K homeostasis, which is necessary for efficient photosynthetic system functioning and stomatal opening regulation [67]. Potassium plays a significant role in plant salinity resistance. Therefore, large quantities are required to reduce osmotic stress in a saline environment [68].

In response to salt stress, it is well established that osmolytes such as organic and inorganic solutes regulate the cellular osmotic potential of plants. The presence of more of these compounds aids in the selection of stress-tolerant cultivars [69,70]. The increased proline and soluble sugar contents protect cells from salt stress by maintaining the osmotic potential and ionic balance in the cytosol and outside of the cell, resulting in increased water and mineral absorption and cell membrane stability [71]. These increases are also commonly used to protect and stabilize enzyme structures against ROS [5,7]. Under salt stress, the proline content increased significantly in the current study. The present findings are consistent with previous research on *Brassica species* [44], *Phaseolus vulgaris* [72], and *Xanthoceras sorbifoliu* [65], which revealed an increase in proline content under salt stress. The enhancement of the proline content might be due to increased activity of the pyrroline-5-carboxylate synthase (P5CS) of the proline biosynthetic pathway in *Hibiscus cannabinus* under salt stress. Higher enzymatic activities, which aid in regulating cellular structures and functions via interactions with macromolecules, could explain the increased total soluble sugars [73,74]. The current study found a significant increase in the total soluble sugar when exposed to salinity stress, consistent with [75,76]. Furthermore, soluble proteins act as osmotin, and their accumulation may play a role in the development of salt tolerance [6]. The current study revealed that under salt stress, the protein content increased significantly. The present findings are consistent with previous studies [6,7].

Salt stress may disrupt the proper balance between the induced ROS production and elimination, resulting in oxidative stress. Excessive levels of radical species, such as H_2_O_2_, O_2_^•−^, and OH, damage plant cell components, resulting in cell death [77,78]. This study investigated the redox state of *Hibiscus cannabinus* seedlings by measuring the H_2_O_2_ and O_2_^•−^ levels. The levels of hydrogen peroxide and superoxide anion increased significantly as the NaCl concentration increased. Abiotic-stress-induced increases in ROS generation often peroxidize cellular and organelle membrane lipids, resulting in membrane integrity losses [79]. MDA is commonly used to detect lipid peroxidation. It is a marker of oxidative damage induced by salinity stress, and the higher the level of MDA under stress, the greater the degree of membrane damage [69]. In this study, the MDA content under stress was significantly higher compared to the control. Similarly, a previous study found that increasing the NaCl concentration increased the MDA level in *H. cannabinus* [79]. Plants have a variety of defense systems against the harmful effects of oxygen radicals, including osmolytes and antioxidants [7,44].

Plants produce antioxidants such as phenolic and flavonoid compounds to scavenge or detoxify ROS [80]. In salt-exposed plants, the biosynthesis of such compounds is generally stimulated [81]. In this study, the phenolics and flavonoids increased significantly in salt-exposed seedlings compared to the control. Salinity alters the biosynthesis of primary and secondary metabolites in plants, as previously demonstrated in *Carthamus tinctorius* [82] and maize [83]. The increased phytochemicals with antioxidant properties in salt-stressed *H. cannabinus* improved the defense systems necessary to detoxify or prevent the detrimental effect of the increased production of ROS that occurs with stress conditions.

Alterations in the saponin content are reported in many plants subjected to salinity stress [84]. In this study, the saponin content was highest in plants treated with 200 mM of NaCl compared to the control, which then declined significantly with 250 mM of NaCl. Similarly, Mar and colleagues [85] found that the saponins level in *C. quinoa* treated with 200 mM of NaCl increased. Furthermore, the saponin content of cucumber increased with low and moderate salinity levels but decreased significantly with the highest concentration of salt [84]. The total saponin content showed changes under salt treatment, revealing the possible involvement of these compounds in the response of *Hibiscus cannabinus* to salt stress.

Previously, a GC-MS analysis of kenaf leaf hexane extract revealed 13 phytoconstituents [32]. The GC-MS analysis of the kenaf leaves revealed 19 compounds in our study. However, when exposed to salt stress, the relative percentages of the compounds changed significantly. The proportions of phytol decreased as the salt concentrations increased and then disappeared at higher levels of salinity stress. Phytol has antioxidant, antibacterial, anti-inflammatory, neuroprotective, analgesic, and anticancer properties [86]. The production of phthalic acid, heptyl undecyl ester, and β-monoolein disappeared under salt stress. The production of 1-heptacosanol was stimulated at low salinity but disappeared after the severe salt treatment. There have been several reports on the antibacterial and antioxidant activity of 1-heptacosanol [87,88]. Furthermore, a low level of salinity stress stimulated the production of oleic acid, 3-(octadecyloxy) propyl ester. However, a high level of salinity stress did not notably change the level of its content. Oleic acid, 3-(octadecyloxy) propyl ester has potent antifungal activity [89]. Some new compounds appeared under the salt stress, such as β-viscol, diisooctyl phthalate, and 3,7,11,15-tetramethyl-2E,6E,10E,14-hexadecatetraenyl acetate. Furthermore, alkanes such as heptacosane and tetratetracontane appeared at the highest salt concentration. These compounds may be responsible for antibacterial, anticancer, antiviral, and antifungal activities [90]. The biosynthesis of terpenes was inhibited and then disappeared with 150 and 200 mM of NaCl. These findings are consistent with a previous study in which salt inhibited terpene biosynthesis. Salt treatment can have an impact on the medicinal properties of *Hibiscus cannabinus*.

The present study revealed significant increases in the DPPH, ABTS, FRAP, and ferrozine antioxidant activity levels of *H. cannabinus* under NaCl stress. A previous study found that salt treatment increased the antioxidant capacity of sea lavender in terms of DPPH, ABTS, and FRAP [91]. Muscolo et al. [63] found that the DPPH, ABTS, FRAP, and ferrozine antioxidant activities in lentils increased significantly under NaCl stress. These antioxidant capacities could be associated with phenolic compound levels [92]. Similarly, the current study found that the antioxidant activity of *H. cannabinus* extracts was strongly related to the total phenolic content. The RACI and chemical assay results correlated, suggesting that the RACI could be used to measure food antioxidant power levels [93]. The salinity stress, thus, had a significant impact on the antioxidant capacity of the plant extracts. It could be indicated as a suitable strategy to increase the antioxidant activities of medicinal plants.

## 5. Conclusions

In *H. cannabinus*, the NaCl treatment increased the proline, soluble sugars, soluble protein, total phenolic content, and total flavonoid contents while lowering plant growth, K, Ca, Mg, and P levels. More osmolytes and antioxidants may improve its resistance to salinity stress. In addition, the NaCl treatment significantly enhanced the antioxidant capacity. The salt stress significantly affected the constituents of volatile compounds in *H. cannabinus*. The production of 1-heptacosanol was stimulated at low salinity but disappeared after the severe salt treatment. Moreover, the salt stress induced the biosynthesis of new compounds such as heptacosane, 1-octadecanesulphonyl chloride, and tetratetracontane. Therefore, the emergence of chemotypes at various salt concentrations may be an advantageous consequence of salinity stress in some plants, causing them to create substances with industrial and therapeutic significance.

## Figures and Tables

**Table 1:** Compositions of nitrogen, potassium, calcium, magnesium, phosphorous, and iron in the leaves of *H. cannabinus* subjected to different levels of salt stress (0, 50, 100, 150, 200, and 250 mM of NaCl). The results are expressed in mg/g dry weight (DW), as means ± SD (*n* = 3). According to the Duncan test (*p* < 0.05), different letters within a column indicate significant differences.

Treatments (mM)	N (mg/g DW)	K (mg/g DW)	Ca (mg/g DW)	Mg (mg/g DW)	*p* (mg/g DW)	Fe (mg/g DW)
0	63.07 ± 0.04 ^a^	29.65 ± 0.22 ^a^	9.59 ± 0.32 ^a^	1.14 ± 0.01 ^a^	3.59 ± 0.05 ^a^	0.13 ± 0.00 ^b^
50	62.13 ± 0.04 ^a^	27.72 ± 0.02 ^d^	7.38 ± 0.14 ^b^	1.08 ± 0.01 ^a, b^	3.31 ± 0.03 ^b^	0.17 ± 0.00 ^a^
100	57.43 ± 0.45 ^b^	29.00 ± 0.19 ^a, b^	5.26 ± 0.06 ^c^	1.01 ± 0.03 ^b^	2.80 ± 0.09 ^c^	0.10 ± 0.00 ^c^
150	53.40 ± 1.33 ^c^	28.87 ± 0.90 ^a–c^	4.15 ± 0.13 ^d^	0.90 ± 0.07 ^c^	2.10 ± 0.05 ^d^	0.09 ± 0.00 ^c^
200	48.80 ± 0.36 ^d^	27.95 ± 0.10 ^c, d^	2.78 ± 0.04 ^e^	0.86 ± 0.00 ^c^	2.05 ± 0.05 ^d^	0.08 ± 0.00 ^c^
250	47.40 ± 0.46 ^e^	28.50 ± 0.09 ^b–d^	5.10 ± 0.06 ^c^	0.88 ± 0.02 ^c^	2.16 ± 0.07 ^d^	0.09 ± 0.00 ^c^

**Table 2:** Changes in the compositions and relative percentages (%) of volatile compounds in the leaves of *H. cannabinus* under different NaCl concentrations (0, 50, 100, 150, 200, and 250 mM). The results, reported as percentages, are means ± SD (*n* = 3). According to the Duncan test (*p* < 0.05), different letters within a row differ significantly; nd, not detected.

No.	Name of the Compound	Content (%)					
		NaCl Concentration (mM)					
		0	50	100	150	200	250
1	Methyl linolelaidate	4.36 ± 0.49 ^b^	3.43 ± 0.30 ^c^	2.92 ± 0.36 ^d^	9.15 ± 0.68 ^a^	1.87 ± 0.19 ^e^	nd
2	Phytol	18.64 ± 1.60 ^a^	2.32 ± 0.45 ^c^	2.09 ± 0.15 ^d^	5.73 ± 0.10 ^b^	nd	nd
3	Oleamide	9.36 ± 0.64 ^c^	10.86 ± 0.10 ^b^	5.02 ± 0.07 ^f^	16.32 ± 1.18 ^a^	7.92 ± 0.1 ^d^	6.12 ± 0.05 ^e^
4	Alterungsschutzmittel BKF	7.30 ± 0.74 ^b^	6.25 ± 0.15 ^c^	2.40 ± 0.17 ^e^	10.30 ± 0.15 ^a^	5.01 ± 0.20 ^d^	2.06 ± 0.03 ^d^
5	Diisooctyl phthalate	2.52 ± 0.70 ^c^	5.25 ± 0.73 ^a^	0.96 ± 0.14 ^d^	Nd	4.10 ± 0.89 ^b^	3.08 ± 0.23 ^c^
6	Phthalic acid, heptyl undecyl ester	6.70 ± 1.38 ^d^	6.55 ± 0.12 ^e^	68.50 ± 2.50 ^a^	11.43 ± 0.59 ^b^	9.96 ± 1.54 ^c^	4.76 ± 0.44 ^f^
7	Heptacosane	nd	nd	nd	Nd	1.55 ± 0.91 ^b^	2.78 ± 0.66 ^a^
8	1-Octadecanesulphonyl chloride	nd	nd	nd	Nd	0.34 ± 0.14	nd
9	α-Glyceryl linolenate	2.72 ± 0.10 ^c^	23.36 ± 0.94 ^b^	0.68 ± 0.04 ^d^	0.29 ± 0.06 ^e^	nd	42.85 ± 1.10 ^a^
10	β-Monoolein	2.06 ± 0.20 ^c^	4.60 ± 0.14 ^b^	0.57 ± 0.10 ^d^	13.96 ±1.50 ^a^	nd	0.55 ± 0.12 ^e^
11	1-Heptacosanol	18.23 ±1.92 ^b^	3.08 ± 0.32 ^e^	4.61 ± 0.34 ^d^	Nd	19.47 ± 1.44 ^a^	13.97 ± 0.95 ^c^
12	12-Methyl-E,E-2,13-octadecadien-1-ol	8.27 ± 0.15 ^b^	20.73 ± 2.00 ^a^	2.90 ± 0.52 ^d^	Nd	4.35 ± 0.16 ^c^	1.84 ± 0.15 ^e^
13	Oleic acid, 3-(octadecyloxy)propyl ester	3.57 ± 0.03 ^e^	6.16 ± 0.86 ^c^	5.98 ± 0.12 ^d^	31.67 ± 1.56 ^b^	40.02 ± 0.10 ^a^	2.87 ± 0.56 ^f^
14	cis-Vaccenic acid	6.80 ± 0.25 ^a^	1.64 ± 0.14 ^b^	0.57 ± 0.08 ^d^	1.16 ± 0.04 ^c^	nd	nd
15	Tetratetracontane	nd	nd	nd	nd	0.71 ± 0.14 ^b^	0.89 ± 0.59 ^a^
16	Ethyl iso-allocholate	1.81 ± 0.03 ^a^	0.50 ± 0.01 ^b^	0.23 ± 0.09 ^c^	nd	nd	0.15 ± 0.04 ^d^
17	β-Sitosterol	0.92 ± 0.04 ^c^	2.13 ± 0.13 ^b^	0.22 ± 0.01 ^e^	nd	0.34 ± 0.05 ^d^	9.48 ± 0.50 ^a^
18	β-Viscol	1.45 ± 0.05 ^a^	1.12 ± 0.15 ^b^	0.28 ± 0.02 ^c^	nd	0.15 ± 0.02 ^e^	0.16 ± 0.02 ^d^
19	Unknown	3.90 ± 0.15 ^b^	0.43 ± 0.04 ^e^	1.93 ± 0.15 ^d^	nd	3.32 ± 0.18 ^c^	5.07 ± 0.25 ^a^
20	Unknown	1.39 ± 0.14 ^c^	1.60 ± 0.24 ^b^	0.15 ± 0.05 ^e^	nd	0.89 ± 0.14 ^d^	3.62 ± 0.19 ^a^
	Total	100	100	100	100	99.90	100
	Total identified classes						
	Alkanes	0.00 ^c^	0.00 ^c^	0.00 ^c^	0.00 ^c^	2.60 ± 0.21 ^b^	3.67 ± 3.25 ^a^
	Esters	19.41 ± 1.78 ^f^	44.10 ± 2.62 ^e^	78.65 ± 0.48 ^a^	66.50 ± 2.24 ^b^	51.85 ± 0.99 ^c^	51.03 ± 0.79 ^d^
	Phenols	7.30 ± 0.74 ^b^	6.25 ± 0.15 ^c^	2.40 ± 0.17 ^e^	10.30 ± 0.15 ^a^	5.01 ± 0.61 ^d^	1.91 ± 0.03 ^f^
	Amide	9.36 ± 0.64 ^c^	10.86 ± 0.10 ^b^	5.02 ± 0.07 ^f^	16.32 ± 1.18 ^a^	7.92 ± 0.10 ^d^	6.12 ± 0.01 ^e^
	Alcohols	27.42 ± 1.06 ^a^	25.94 ± 1.13 ^b^	7.73 ± 0.12 ^e^	0.00 ^f^	24.16 ± 1.61 ^d^	25.29 ± 0.21 ^c^
	Terpenes	20.09 ± 1.60 ^a^	3.44 ± 0.45 ^c^	2.37 ± 0.15 ^d^	5.73 ± 0.42 ^b^	0.15 ± 0.001 ^e^	0.16 ± 0.002 ^f^
	Steroid derivative	1.81 ± 0.03 ^a^	0.50 ± 0.01 ^b^	0.23 ± 0.09 ^c^	0.00 ^e^	0.00 ^e^	0.15 ± 0.003 ^d^
	Others	14.61 ± 0.20 ^a^	8.92 ± 0.14 ^c^	3.61 ± 0.04 ^e^	1.16 ± 0.06 ^f^	8.31 ± 0.02 ^d^	11.77 ± 0.14 ^b^

## Data Availability

Not applicable.

## References

[1] Kashif M.H., Tang D., Li Z., Wei F., Liang Z., Chen P. (2020). Comparative Cytological and Gene Expression Analysis Reveals Potential Metabolic Pathways and Target Genes Responsive to Salt Stress in Kenaf (*Hibiscus cannabinus* L.). J. Plant Growth Regul..

[2] Munns R., Day D.A., Fricke W., Watt M., Arsova B., Barkla B.J., Bose J., Byrt C.S., Chen Z., Foster K.J. (2020). Energy Costs of Salt Tolerance in Crop Plants. New Phytol..

[3] Liu M., Pan T., Allakhverdiev S.I., Yu M., Shabala S. (2020). Crop Halophytism: An Environmentally Sustainable Solution for Global Food Security. Trends Plant Sci..

[4] Li-ping L., Xiao-hua L., Hong-bo S., Zhao-Pu L., Ya T., Quan-suo Z., Jun-qin Z. (2015). Ameliorants Improve Saline–Alkaline Soils on a Large Scale in Northern Jiangsu Province, China. Ecol. Eng..

[5] Rahneshan Z., Nasibi F., Moghadam A.A. (2018). Effects of Salinity Stress on Some Growth, Physiological, Biochemical Parameters and Nutrients in Two Pistachio (*Pistacia vera* L.) Rootstocks. J. Plant Interact..

[6] Zhang M., Fang Y., Ji Y., Jiang Z., Wang L. (2013). Effects of Salt Stress on Ion Content, Antioxidant Enzymes and Protein Profile in Different Tissues of *Broussonetia papyrifera*. S. Afr. J. Bot..

[7] Kumar S., Li G., Yang J., Huang X., Ji Q., Liu Z., Ke W., Hou H. (2021). Effect of Salt Stress on Growth, Physiological Parameters, and Ionic Concentration of Water Dropwort (*Oenanthe javanica*) Cultivars. Front. Plant Sci..

[8] Singh M. (2014). Plant Tolerance Mechanism Against Salt Stress: The Nutrient Management Approach. Biochem. Pharm..

[9] Rahman A., Hossain M.S., Mahmud J.-A., Nahar K., Hasanuzzaman M., Fujita M. (2016). Manganese-Induced Salt Stress Tolerance in Rice Seedlings: Regulation of Ion Homeostasis, Antioxidant Defense and Glyoxalase Systems. Physiol. Mol. Biol. Plants.

[10] Ali Q., Daud M.K., Haider M.Z., Ali S., Rizwan M., Aslam N., Noman A., Iqbal N., Shahzad F., Deeba F. (2017). Seed Priming by Sodium Nitroprusside Improves Salt Tolerance in Wheat (*Triticum aestivum* L.) by Enhancing Physiological and Biochemical Parameters. Plant Physiol. Biochem..

[11] Sahin U., Ekinci M., Ors S., Turan M., Yildiz S., Yildirim E. (2018). Effects of Individual and Combined Effects of Salinity and Drought on Physiological, Nutritional and Biochemical Properties of Cabbage (*Brassica oleracea* Var *Capitata*). Sci. Hortic..

[12] Nxele X., Klein A., Ndimba B.K. (2017). Drought and Salinity Stress Alters ROS Accumulation, Water Retention, and Osmolyte Content in Sorghum Plants. S. Afr. J. Bot..

[13] Rahman M.M., Mostofa M.G., Das A.K., Anik T.R., Keya S.S., Ahsan S.M., Khan M.A.R., Ahmed M., Rahman M.A., Hossain M.M. (2022). Ethanol Positively Modulates Photosynthetic Traits, Antioxidant Defense and Osmoprotectant Levels to Enhance Drought Acclimatization in Soybean. Antioxidants.

[14] De Rossi S., Di Marco G., Bruno L., Gismondi A., Canini A. (2021). Investigating the Drought and Salinity Effect on the Redox Components of *Sulla coronaria* (L.) Medik. Antioxidants.

[15] Shahid M.A., Sarkhosh A., Khan N., Balal R.M., Ali S., Rossi L., Gómez C., Mattson N., Nasim W., Garcia-Sanchez F. (2020). Insights into the Physiological and Biochemical Impacts of Salt Stress on Plant Growth and Development. Agronomy.

[16] Chen C., Wang C., Liu Z., Liu X., Zou L., Shi J., Chen S., Chen J., Tan M. (2018). Variations in Physiology and Multiple Bioactive Constituents under Salt Stress Provide Insight into the Quality Evaluation of Apocyni Veneti Folium. Int. J. Mol. Sci..

[17] Pang J., Cuin T., Shabala L., Zhou M., Mendham N., Shabala S. (2007). Effect of Secondary Metabolites Associated with Anaerobic Soil Conditions on Ion Fluxes and Electrophysiology in Barley Roots. Plant Physiol..

[18] Hasanuzzaman M., Bhuyan M.H.M., Zulfiqar F., Raza A., Mohsin S., Mahmud J., Fujita M., Fotopoulos V. (2020). Reactive Oxygen Species and Antioxidant Defense in Plants under Abiotic Stress: Revisiting the Crucial Role of a Universal Defense Regulator. Antioxidants.

[19] Sachdev S., Ansari S.A., Ansari M.I., Fujita M., Hasanuzzaman M. (2021). Abiotic Stress and Reactive Oxygen Species: Generation, Signaling, and Defense Mechanisms. Antioxidants.

[20] Akram N.A., Shafiq F., Ashraf M. (2017). Ascorbic Acid-A Potential Oxidant Scavenger and Its Role in Plant Development and Abiotic Stress Tolerance. Front. Plant Sci..

[21] Chen W., Feng C., Guo W., Shi D., Yang C. (2011). Comparative Effects of Osmotic-, Salt- and Alkali Stress on Growth, Photosynthesis, and Osmotic Adjustment of Cotton Plants. Photosynthetica.

[22] Kobaisy M., Tellez M.R., Webber C.L., Dayan F.E., Schrader K.K., Wedge D.E. (2001). Phytotoxic and Fungitoxic Activities of the Essential Oil of Kenaf (*Hibiscus cannabinus* L.) Leaves and Its Composition. J. Agric. Food Chem..

[23] Zhou Y., Tang N., Huang L., Zhao Y., Tang X., Wang K. (2018). Effects of Salt Stress on Plant Growth, Antioxidant Capacity, Glandular Trichome Density, and Volatile Exudates of Schizonepeta Tenuifolia Briq. Int. J. Mol. Sci..

[24] Rubio M.C., Bustos-Sanmamed P., Clemente M.R., Becana M. (2009). Effects of Salt Stress on the Expression of Antioxidant Genes and Proteins in the Model Legume *Lotus Japonicus*. New Phytol..

[25] Li H. (2017). RNA-Seq for Comparative Transcript Profiling of Kenaf under Salinity Stress. J. Plant Res..

[26] Kim D.-G., Ryu J., Lee M.-K., Kim J.M., Ahn J.-W., Kim J.-B., Kang S.-Y., Bae C.-H., Kwon S.-J. (2018). Nutritional Properties of Various Tissues from New Kenaf Cultivars. J. Crop Sci. Biotechnol..

[27] Lin D., Xiao M., Zhao J., Li Z., Xing B., Li X., Kong M., Li L., Zhang Q., Liu Y. (2016). An Overview of Plant Phenolic Compounds and Their Importance in Human Nutrition and Management of Type 2 Diabetes. Molecules.

[28] Maganha E.G., da Costa Halmenschlager R., Rosa R.M., Henriques J.A., de Paula Ramos A.L., Saffi J. (2010). Pharmacological Evidences for the Extracts and Secondary Metabolites from Plants of the Genus *Hibiscus*. Food Chem..

[29] Xie P., Huang L., Zhang C., Ding S., Deng Y., Wang X. (2018). Skin-Care Effects of Dandelion Leaf Extract and Stem Extract: Antioxidant Properties, Tyrosinase Inhibitory and Molecular Docking Simulations. Ind. Crops Prod..

[30] Sim Y.Y., Jess Ong W.T., Nyam K.L. (2019). Effect of Various Solvents on the Pulsed Ultrasonic Assisted Extraction of Phenolic Compounds from *Hibiscus cannabinus* L. Leaves. Ind. Crops Prod..

[31] Zhao S., Li X., Cho D., Arasu M., Al-Dhabi N., Park S. (2014). Accumulation of Kaempferitrin and Expression of Phenyl-Propanoid Biosynthetic Genes in Kenaf (*Hibiscus cannabinus*). Molecules.

[32] Ryu J., Kwon S.-J., Ahn J.-W., Jo Y.D., Kim S.H., Jeong S.W., Lee M.K., Kim J.-B., Kang S.-Y. (2017). Phytochemicals and Antioxidant Activity in the Kenaf Plant (*Hibiscus cannabinus* L.). J. Plant Biotechnol..

[33] Survay N. (2011). New Genera of Flavonols and Flavonol Derivatives As Therapeutic Molecules. J. Korean Soc. Appl. Biol. Chem..

[34] Byju K., Vasundhara G., Anuradha V., Nair S.M., Kumar N.C. (2013). Presence of Phytol, a Precursor of Vitamin E in Chaetomorpha Antinnina. Mapana J. Sci..

[35] Da-Costa-Rocha I., Bonnlaender B., Sievers H., Pischel I., Heinrich M. (2014). *Hibiscus sabdariffa* L.—A Phytochemical and Pharmacological Review. Food Chem..

[36] Rajaram S. (2014). Health Benefits of Plant-Derived α-Linolenic Acid. Am. J. Clin. Nutr..

[37] Agbor G., Oben J., Blaise N., Takala J., Jeanne N. (2005). Hepatoprotective Activity of *Hibiscus cannabinus* (Linn.) Against Carbon Tetrachloride and Paracetamol Induced Liver Damage in Rats. Pak. J. Biol. Sci..

[38] Pradeep K., Gagandeep K., Nanjaian M. (2010). Antihyperlipidemic Effect of Hydroalcoholic Extract of Kenaf (*Hibiscus cannabinus* L.) Leaves in High Fat Diet Fed Rats. Ann. Biol. Res..

[39] Lee Y.G., Byeon S.E., Kim J.Y., Lee J.Y., Rhee M.H., Hong S., Wu J.C., Lee H.S., Kim M.J., Cho D.H. (2007). Immunomodulatory Effect of Hibiscus Cannabinus Extract on Macrophage Functions. J. Ethnopharmacol..

[40] Brunetti C., Di Ferdinando M., Fini A., Pollastri S., Tattini M. (2013). Flavonoids as Antioxidants and Developmental Regulators: Relative Significance in Plants and Humans. Int. J. Mol. Sci..

[41] Jin C.H., Ghimeray A., Wang L., Xu M.L., Piao J.P., Cho D.H. (2013). Far Infrared Assisted Kenaf Leaf Tea Preparation and Its Effect on Phenolic Compounds, Antioxidant and ACE Inhibitory Activity. J. Med. Plant Res..

[42] Munns R., Tester M. (2008). Mechanisms of Salinity Tolerance. Annu. Rev. Plant Biol..

[43] Bistgani Z.E., Hashemi M., DaCosta M., Craker L., Maggi F., Morshedloo M.R. (2019). Effect of Salinity Stress on the Physiological Characteristics, Phenolic Compounds and Antioxidant Activity of *Thymus vulgaris* L. and Thymus Daenensis Celak. Ind. Crops Prod..

[44] El-Badri A.M., Batool M., A. A. Mohamed I., Wang Z., Khatab A., Sherif A., Ahmad H., Khan M.N., Hassan H.M., Elrewainy I.M. (2021). Antioxidative and Metabolic Contribution to Salinity Stress Responses in Two Rapeseed Cultivars during the Early Seedling Stage. Antioxidants.

[45] Wang C., Chen L., Cai Z., Chen C., Liu Z., Liu X., Zou L., Chen J., Tan M., Wei L. (2019). Dynamic Variations in Multiple Bioactive Constituents under Salt Stress Provide Insight into Quality Formation of Licorice. Molecules.

[46] Deng Y., Li D., Huang Y., Huang S. (2017). Physiological Response to Cadmium Stress in Kenaf (*Hibiscus cannabinus* L.) Seedlings. Ind. Crops Prod..

[47] Wei F., Tang D., Li Z., Kashif M.H., Khan A., Lu H., Jia R., Chen P. (2019). Molecular Cloning and Subcellular Localization of Six HDACs and Their Roles in Response to Salt and Drought Stress in Kenaf (*Hibiscus cannabinus* L.). Biol Res..

[48] Hoagland D.R., Arnon D.I. (1950). The Water Culture Method for Growing Plants without Soil. Calif. Agric. Exp. Stn. Circ..

[49] Hseu Z.-Y. (2004). Evaluating Heavy Metal Contents in Nine Composts Using Four Digestion Methods. Bioresour. Technol..

[50] Du Laing G., Tack F.M.G., Verloo M.G. (2003). Performance of Selected Destruction Methods for the Determination of Heavy Metals in Reed Plants (*Phragmites australis*). Anal. Chim. Acta.

[51] Bates L.S., Waldren R.P., Teare I.D. (1973). Rapid Determination of Free Proline for Water-Stress Studies. Plant Soil.

[52] Michel D., Gilles K.A., Hamilton J.K., Rebers P.A., Smith F. (1956). Colorimetric Method for Determination of Sugars and Related Substances. Anal. Chem..

[53] Guy C., Haskell D., Neven L., Klein P., Smelser C. (1992). Hydration-State-Responsive Protein Link Cold and Drought Stress in Spinach. Planta.

[54] Okuda T., Matsuda Y., Yamanaka A., Sagisaka S. (1991). Abrupt Increase in the Level of Hydrogen Peroxide in Leaves of Winter Wheat Is Caused by Cold Treatment. Plant Physiol..

[55] Bu R., Xie J., Yu J., Liao W., Xiao X., Lv J., Wang C., Ye J., Calderón-Urrea A. (2016). Autotoxicity in Cucumber (*Cucumis sativus* L.) Seedlings Is Alleviated by Silicon through an Increase in the Activity of Antioxidant Enzymes and by Mitigating Lipid Peroxidation. J. Plant Biol..

[56] Lang D., Yu X., Jia X., Li Z., Zhang X. (2020). Methyl Jasmonate Improves Metabolism and Growth of NaCl-Stressed Glycyrrhiza Uralensis Seedlings. Sci. Hortic..

[57] Heath R.L., Packer L. (1968). Photoperoxidation in Isolated Chloroplast I. Kinetics and Stoichiometry of Fatty Acid Peroxidation. Arch. Biochem. Biophys..

[58] Chrysargyris A., Panayiotou C., Tzortzakis N. (2016). Nitrogen and Phosphorus Levels Affected Plant Growth, Essential Oil Composition and Antioxidant Status of Lavender Plant (*Lavandula angustifolia* Mill.). Ind. Crops Prod..

[59] He J., Wu Z., Zhang S., Zhou Y., Zhao F., Peng Z., Hu Z. (2014). Optimization of Microwave-Assisted Extraction of Tea Saponin and Its Application on Cleaning of Historic Silks. J. Surfact Deterg..

[60] Brand-Williams W., Cuvelier M.E., Berset C. (1995). Use of a Free Radical Method to Evaluate Antioxidant Activity. LWT-Food Sci. Technol..

[61] Nisca A., Ștefănescu R., Stegăruș D.I., Mare A.D., Farczadi L., Tanase C. (2021). Comparative Study Regarding the Chemical Composition and Biological Activity of Pine (*Pinus nigra* and *P. sylvestris*) Bark Extracts. Antioxidants.

[62] Benzie I.F.F., Strain J.J. (1996). The Ferric Reducing Ability of Plasma (FRAP) as a Measure of “Antioxidant Power”: The FRAP Assay. Anal. Biochem..

[63] Muscolo A., Calderaro A., Papalia T., Settineri G., Mallamaci C., Panuccio M.R. (2020). Soil Salinity Improves Nutritional and Health Promoting Compounds in Three Varieties of Lentil (*Lens Culinaris* Med.). Food Biosci..

[64] James R.A., Blake C., Byrt C.S., Munns R. (2011). Major Genes for Na+ Exclusion, Nax1 and Nax2 (Wheat HKT1;4 and HKT1;5), Decrease Na+ Accumulation in Bread Wheat Leaves under Saline and Waterlogged Conditions. J. Exp. Bot..

[65] Zong J.-W., Zhang Z.-L., Huang P.-L., Chen N.-Y., Xue K.-X., Tian Z.-Y., Yang Y.-H. (2021). Growth, Physiological, and Photosynthetic Responses of Xanthoceras Sorbifolium Bunge Seedlings Under Various Degrees of Salinity. Front. Plant Sci..

[66] Oueslati S., Karray-Bouraoui N., Attia H., Rabhi M., Ksouri R., Lachaal M. (2010). Physiological and Antioxidant Responses of Mentha Pulegium (Pennyroyal) to Salt Stress. Acta Physiol Plant.

[67] Bettaieb Rebey I., Bourgou S., Rahali F.Z., Msaada K., Ksouri R., Marzouk B. (2017). Relation between Salt Tolerance and Biochemical Changes in Cumin (*Cuminum cyminum* L.) Seeds. J. Food Drug Anal..

[68] Benito B., Haro R., Amtmann A., Cuin T., Dreyer I. (2014). The Twins K+ and Na+ in Plants?. J. Plant Physiol..

[69] Shabala S., Pottosin I. (2014). Regulation of Potassium Transport in Plants under Hostile Conditions: Implications for Abiotic and Biotic Stress Tolerance. Physiol Plant..

[70] Mahouachi J. (2018). Long-Term Salt Stress Influence on Vegetative Growth and Foliar Nutrient Changes in Mango (*Mangifera indica* L.) Seedlings. Sci. Hortic..

[71] Sarker U., Oba S. (2020). The Response of Salinity Stress-Induced A. Tricolor to Growth, Anatomy, Physiology, Non-Enzymatic and Enzymatic Antioxidants. Front. Plant Sci..

[72] Zulfiqar F., Akram N.A., Ashraf M. (2020). Osmoprotection in Plants under Abiotic Stresses: New Insights into a Classical Phenomenon. Planta.

[73] Gupta B., Huang B. (2014). Mechanism of Salinity Tolerance in Plants: Physiological, Biochemical, and Molecular Characterization. Int. J. Genom..

[74] Al Hassan M., Morosan M., López-Gresa M., Prohens J., Vicente O., Boscaiu M. (2016). Salinity-Induced Variation in Biochemical Markers Provides Insight into the Mechanisms of Salt Tolerance in Common (*Phaseolus vulgaris*) and Runner (*P. coccineus*) Beans. Int. J. Mol. Sci..

[75] Sharif P., Seyedsalehi M., Paladino O., Van Damme P., Sillanpää M., Sharifi A.A. (2018). Effect of Drought and Salinity Stresses on Morphological and Physiological Characteristics of Canola. Int. J. Environ. Sci. Technol..

[76] Ibrahimova U.F. (2019). The Effect of Nacl on Some Physiological and Biochemical Parameters in *Triticum aestivum* L. Genotypes. Plant Physiol..

[77] El-Badri A.M.A. (2021). Modulation of Salinity Impact on Early Seedling Stage via Nano-Priming Application of Zinc Oxide on Rapeseed (*Brassica napus* L.). Plant Physiol. Biochem..

[78] Alam P., Albalawi T.H., Altalayan F.H., Bakht A., Ahanger M.A., Raja V., Ashraf M., Ahmad P. (2019). 24-Epibrassinolide (EBR) Confers Tolerance against NaCl Stress in Soybean Plants by Up-Regulating Antioxidant System, Ascorbate-Glutathione Cycle, and Glyoxalase System. Biomolecules.

[79] Gill S.S., Tuteja N. (2010). Reactive Oxygen Species and Antioxidant Machinery in Abiotic Stress Tolerance in Crop Plants. Plant Physiol. Biochem..

[80] Apel K., Hirt H. (2004). Reactive oxygen species: Metabolism, Oxidative Stress, and Signal Transduction. Annu. Rev. Plant Biol..

[81] Li Z., Hu Y., Chang M., Kashif M.H., Tang M., Luo D., Cao S., Lu H., Zhang W., Huang Z. (2021). 5-Azacytidine Pre-Treatment Alters DNA Methylation Levels and Induces Genes Responsive to Salt Stress in Kenaf (*Hibiscus cannabinus* L.). Chemosphere.

[82] Petridis A., Therios I., Samouris G., Tananaki C. (2012). Salinity-Induced Changes in Phenolic Compounds in Leaves and Roots of Four Olive Cultivars (*Olea europaea* L.) and Their Relationship to Antioxidant Activity. Environ. Exp. Bot..

[83] Navarro J., Flores P., Garrido C., Martínez V. (2006). Changes in the Contents of Antioxidant Compounds in Pepper Fruits at Different Ripening Stages, as Affected by Salinity. Food Chem..

[84] Salem N., Msaada K., Dhifi W., Limam F., Marzouk B. (2014). Effect of Salinity on Plant Growth and Biological Activities of *Carthamus tinctorius* L. Extracts at Two Flowering Stages. Acta Physiol. Plant.

[85] Panuccio M.R., Chaabani S., Roula R., Muscolo A. (2018). Bio-Priming Mitigates Detrimental Effects of Salinity on Maize Improving Antioxidant Defense and Preserving Photosynthetic Efficiency. Plant Physiol. Biochem..

[86] Abdel-Farid I.B., Marghany M.R., Rowezek M.M., Sheded M.G. (2020). Effect of Salinity Stress on Growth and Metabolomic Profiling of *Cucumis sativus* and *Solanum lycopersicum*. Plants.

[87] Caravaca A.M.G., Iafelice G., Lavini A., Pulvento C., Caboni M., Marconi E. (2012). Phenolic Compounds and Saponins in Quinoa Samples (*Chenopodium Quinoa* Willd.) Grown under Different Saline and Nonsaline Irrigation Regimens. J. Agric. Food Chem..

[88] Rajesh K.D., Vasantha S., Panneerselvam A., Valsala Rajesh N., Jeyathilakan N. (2016). Phytochemical analysis, in vitro antioxidant potential and gas chromatography-mass spectrometry studies of *Dicranopteris linearis*. Asian J. Pharm. Clin. Res..

[89] Faridha Begum I., Mohankumar R., Jeevan M., Ramani K. (2016). GC–MS Analysis of Bio-Active Molecules Derived from Paracoccus Pantotrophus FMR19 and the Antimicrobial Activity Against Bacterial Pathogens and MDROs. Indian J. Microbiol..

[90] Al-Abd N.M., Mohamed Nor Z., Mansor M., Azhar F., Hasan M.S., Kassim M. (2015). Antioxidant, Antibacterial Activity, and Phytochemical Characterization of *Melaleuca Cajuputi* Extract. BMC Complement. Altern. Med..

[91] Abubacker M.N., Kamala Devi P. (2014). In Vitro Antifungal Potentials of Bioactive Compound Oleic Acid, 3-(Octadecyloxy) Propyl Ester Isolated from *Lepidagathis Cristata* Willd. (Acanthaceae) Inflorescence. Asian Pac. J. Trop. Biomed..

[92] Thawabteh A., Juma S., Bader M., Karaman D., Scrano L., Bufo S., Karaman R. (2019). The Biological Activity of Natural Alkaloids against Herbivores, Cancerous Cells and Pathogens. Toxins.

[93] Rodrigues M.J., Soszynski A., Martins A., Rauter A., Neng N., Nogueira J., Varela J., Barreira L., Custódio L. (2015). Unravelling the Antioxidant Potential and the Phenolic Composition of Different Anatomical Organs of the Marine Halophyte *Limonium Algarvense*. Ind. Crops Prod..

